# Cephalic arch stenosis: an analysis of outcome by type of first intervention

**DOI:** 10.1186/s42155-023-00424-4

**Published:** 2024-01-19

**Authors:** Umberto Pisano, Karen Stevenson, Ram Kasthuri, David Kingsmore

**Affiliations:** 1 Radiology Department, Queen Elizabeth University Hospital, NHS Greater Glasgow and Clyde, 1345 Govan Road, Glasgow, G51 4TF UK; 2 Renal Transplant Surgery, Queen Elizabeth University Hospital, NHS Greater Glasgow and Clyde, 1345 Govan Road, Glasgow, G51 4TF UK

**Keywords:** Cephalic arch, Angioplasty, Stent graft

## Abstract

**Background:**

Cephalic arch stenoses (CAS) occur in near 70% of elbow arteriovenous fistulas. Percutaneous transluminal angioplasty (PTA) remains first-line treatment despite documented stent-grafts (SG) efficacy. The study aim is to report long-term outcomes based on initial treatment of CAS.

**Methods:**

Retrospective review of 12-year data in single tertiary centre. Outcomes included technical success, rupture rate, primary patency (PP), dialysis performance; categorical variables assessed via χ^2^ or Fisher’s; nonparametric tests used for skewed data. Kaplan–Meier analysis used for PP and cumulative patency. Cox proportional hazard regression model to assess explanatory variables in PP.

**Results:**

One hundred one brachio- and radiocephalic fistulas with CAS were included. SG as first intervention had higher success than PTA (85% vs 61%, *p* = 0.003). Rupture occurred in 9/85 (10.6%) PTA vs 0% in SG (*p* = 0.046). In a subgroup with poor urea reduction rate (URR), both PTA and SG improved dialysis performance post-intervention (*p* = 0.002). SG demonstrated better PP than PTA (79,73,60% patency at 3, 6, 9 months; versus 71,51,47%; *p* = 0.195) and cumulative patency (73,61,61% at 1, 2, 3 years; versus 60,34,26%; *p* < 0.001). Of the variables analyzed, technical success of PTA was the only discriminating factor (coeff.-1.01; RR 35%, *p* = 0.035). Accesses that underwent secondary stenting performed better than primarily stented CAS (*p* = 0.01).

**Conclusions:**

SG superiority is confirmed in CAS, particularly when angioplasty is unsuccessful. While PTA has short-lived benefits, it can improve dialysis performance. Other than higher success rate, primary CAS stenting did not have advantages compared to post-PTA stenting in our study. Other factors related to inflow, outflow, conduit characteristics are presumed to be involved in access longevity.

## Introduction

Cephalic arch stenosis (CAS) is a common occurrence occurring in up to 70% of brachiocephalic fistulas (BCF [1]) and is recognised as the most common cause of BCF failure [2]. Delto-pectoral fascial compression, presence of valves and variable curvature are among the factors that might contribute to the development of CAS [3]. It is also postulated that high flow leads to low wall shear stresses (WSS) with compensatory neointimal hyperplasia and lumen reduction to restore the local WSS [4–6]. Other factors may include endothelial damage and pro-inflammatory, pro-fibrotic mediators [7] with an additive effect of high levels of circulating urea [8].

Guidelines recommend treatment for CAS causative of > 50% lumen reduction with clinical/physiological abnormalities [6, 9, 10]. Percutaneous transluminal angioplasty (PTA) in the form of a plain balloon angioplasty (PBA) has been the mainstay of endovascular treatment [11], though recurrence is very common, with 23–76% patency at six months, and 9.5–45% at one year [6, 12–17]. Drug covered balloons (DCB) have shown non-inferiority to PBA in RCT settings [18, 19] but long-term data beyond 6 months with regards to the cephalic arch remain limited. Bare metal stents (BMS) have been tried with similar or better patency to PTA [20]. Stent grafts (SG) have been reported to have better primary patency than PTA and BMS [21–23]: vein wall rupture, endothelial flaps or thrombosis related to venous spasm are typically avoided in SG deployment [24, 25]. PTA generally continues to be performed as first-line intervention, and SG are reserved to recurrent cases [26]. This is partially due to the relative lack of patency data of SG beyond 6 months, their substantial cost, and possible encroachment over venous bifurcations, potentially compromising future haemodialysis (HD) access options. While most literature focuses on direct comparison of angioplasty and stenting in CAS, it is not unusual for HD accesses to undergo both procedures at different times. Therefore, the aim of this article was to review the outcomes of angioplasty first to a stent-graft first approach in treating CAS in dysfunctional arteriovenous fistulas (AVF).

## Methods

A retrospective review of data extracted from local radiology procedure database (CRIS *Healthcare Software Solution Limited*, Mansfield, England, UK) and image database (CareStream Vue PACS, Rochester, NY, USA) was performed for the time period 2009 – 2021. Individuals with end stage renal failure, with arteriovenous access and documented symptomatic or clinically significant CAS were identified (Tables 1 and 2). Interventions before and after the index procedure on same patient, on same or different HD access with CAS, were also reviewed and included when appropriate. In our centre referral for vascular access assessment is triggered by a fall in GFR to < 15 ml/min or a trajectory of GFR that will reach 10 ml/min within 6 months. Commencement of haemodialysis is a clinical decision encompassing biochemical parameters, fluid status and uraemic symptoms. Nine interventional radiology consultants with at least 5 years of endovascular practice following completion of training performed the procedures in the study period.

**Table 1:** Patients’ characteristics

**101 HD Accesses, 96 Patients**	
Age (median)	62 (IQR 54—71)
Males	63 (65.6%)
Females	33 (34.4%)
**ESRF Diagnosis in 96 patients**	
Diabetes	35 (36.5%)
ADPCKD (adult dominant polycystic kidney disease)	9 (9.4%)
IgA nephropathy	8 (8.3%)
Lithium nephropathy	7 (7.3%)
Reflux nephropathy	5 (5.2%)
Obstructive nephropathy	4 (4.2%)
Granulomatosis with polyangiitis (former Wegener’s vasculitis)	2 (2.1%)
Other	16 (16.6%)
Unknown	10 (10.4%)

**Table 2:** Access characteristics and events during the study

**101 HD Accesses, 96 Patients**	
Length of renal replacement therapy with index access (median days) prior to CAS treatment	693 (IQR 429 – 1285)
Radiocephalic Arteriovenous Fistula/e	2 (1.9%)
Brachiocephalic Arteriovenous Fistula/e	99 (98.1%)
Follow-up Period (days)	277 (IQR 135 – 920)
**Main indication for first intervention (101 HD Accesses)**	
Raised pressure during dialysis / prolonged puncture site bleeding	56 (55.3%)
Aneurysmal changes in conduit	28 (27.7%)
Poor URR	17 (16.9%)
**Cephalic Arch Stenosis Distribution Pattern in Percentage (101 HD Accesses) **(Fig. 1)	
A – ascending	*8%*
B – apical	*28.2%*
C – confluence	*27.6%*
D – multifocal	*36.2%*
**Events during the Study (192 interventions)**	
Access thrombosis	12 (6.3%)
Switch to different arteriovenous HD access	36 (18.8%)
TCVC insertion	22 (11.4%)

All angioplasty and SG insertion procedures in the time period were performed with conventional two-dimensional digital subtraction angiography (DSA) and a CAS was addressed over a crossing 0.035’’ wire in all cases. A symptomatic (Table 2) CAS seen to have at least 50% lumen reduction on DSA would have been presumed to merit treatment.

Clinical and dialysis data related to the access of interest was extracted from the regional renal database (*Scottish Electronic Renal Patient Record*, ‘SERPR’). Data analysis was performed using SPSS (*Statistical Product and Service Solutions,* version 28*,* IBM, New York, USA).

**Fig. 1:**
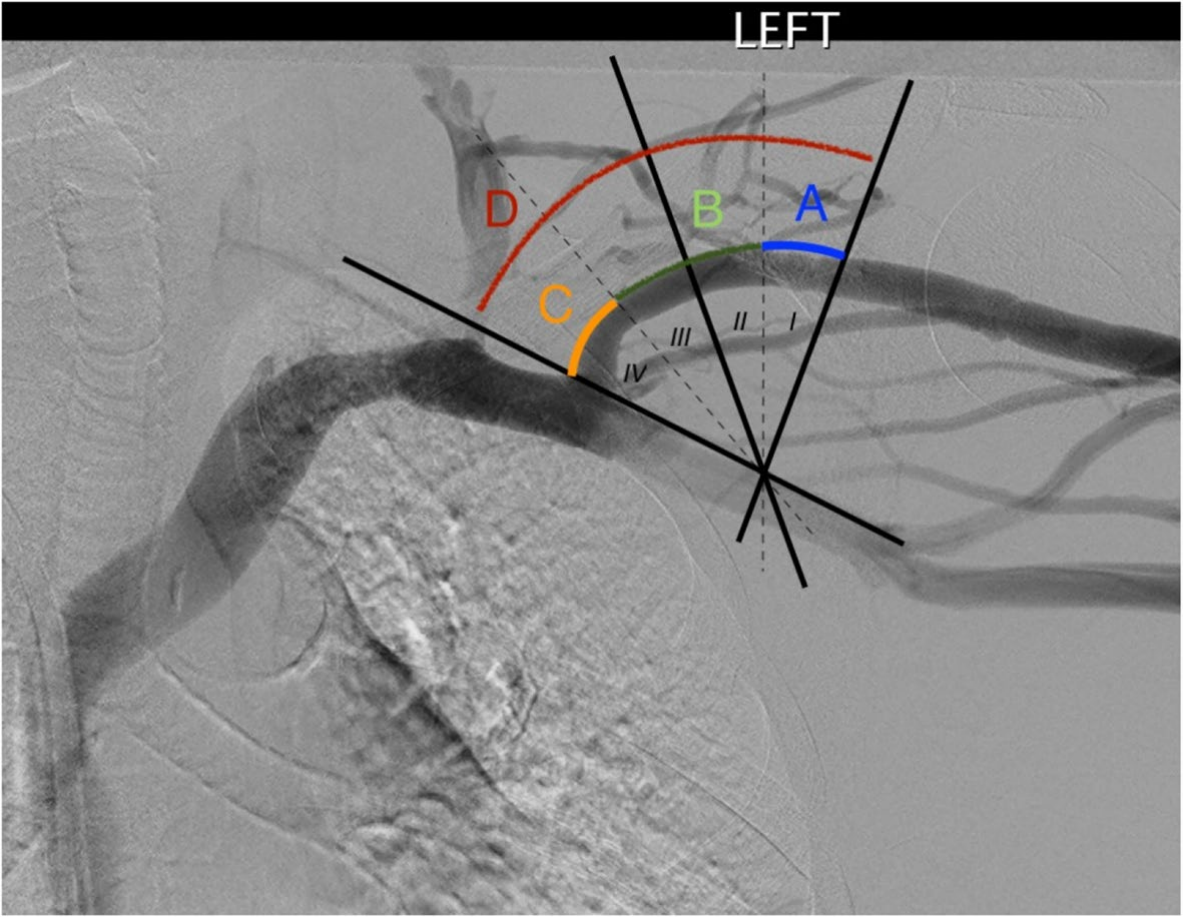
Division of the cephalic arch into four domains in roman numerals (as per Bennett et al. [2]) and proposed modification (in colours). Domains II and III are combined into a single segment. The boundaries of domains I and IV are left unchanged. CAS coding is defined as **A** (*ascending* – domain I), **B** (*apex*, domains II and III), **C** (*confluence*, domain IV). Multidomain involvement is coded as **D**

Data included the age at the time of intervention, gender, main diagnosis, type of access and date of creation; semiquantitative stenosis estimate (25–50%, 50–75%, > 75%), absence/presence of duplicate cephalic arch (CA), angulation of CA in relation to its apex (i.e., *‘alpha angle’*) and localisation of the stenosis within the CA domains: this was modified from the original description by Bennett et al. [2] (Fig. 1)*.* In real world settings, appearances and angulation of the CA can be altered by several factors, including the orientation of C-arm, the level of abduction of the upper limb, and the tortuosity of the CA that could preclude precise identification of the apex*:* hence, segments II and III are fused into a single domain B.

Variables related to the balloon intervention included technical success (< 30% residual stenosis), type and size of the balloon, and whether rupture occurred. For SG, type and make, length and diameter, angulation of the CA post-deployment, whether the SG deployment was into deep venous system (i.e., > 1cm entering the subclavian segment), relationship between stent size and native CA diameter (S/V ratio). The outcomes of the study were as follows:

Primary patency (PP) [27], inclusive of both the index cephalic arch segment and whole conduit, was recorded. If functional PP was lost, the clinical outcome was recorded as a switch to another arteriovenous (AV) access, a tunnelled central venous catheter (TCVC) insertion or peritoneal dialysis. Death and renal transplant were treated as censored events.

Rate of technical success (< 30% residual stenosis after angioplasty or stent), and the rate of rupture (both defined radiologically). Also, the combined time interval(s) between the index and following intervention (Post-intervention Assisted Primary Patency, PAPP) [27] to estimate the overall cumulative patency. Before-and-after Urea Reduction Rates (URR) were estimated for each intervention when applicable.

### Statistical analysis

Analysis between groups of categorical variables was performed via Continuity correction χ^2^ or Fisher’s exact test, when appropriate. After ascertaining skewed data distribution not amenable to transformation, non-parametric tests were used to assess variability in distribution between groups. Calculation of PP of angioplasty-first and SG-first groups as well as PAPP / cumulative patency was estimated using a Kaplan–Meier Log-rank survival test. A multi-variate proportional hazard Cox regression with enter model was created for PP using explanatory variables. A *p* value of < 0.05 was considered significant.

## Results

CAS was identified in 101 AV accesses in 96 patients; 63 patients (65.6%) were males, 35 (36.5%) were diabetic. A total of 192 interventions were performed. 5 arteriovenous grafts (1 axillo-cephalic and 4 brachiocephalic grafts, which underwent 11 interventions) were excluded. BMS insertions and angioplasties with different balloons (DCB, cutting balloons, scoring balloons, high-pressure balloons) other than PBA were also excluded to maximise data homogeneity.

The average follow-up period was 517 days (median 277, IQR 135 – 920), for a total of 3913 days. Access thrombosis occurred in 12 cases (6.3% of interventions), a switch to alternative arteriovenous access in 36 (18.8%) cases and the insertion of a TCVC in 22 (11.4%) cases. Death (not access-related) occurred in 12 cases; 7 patients underwent successful renal transplantation. Characteristics of patients and HD accesses are provided in Tables 1 and 2.

**Fig. 2:**
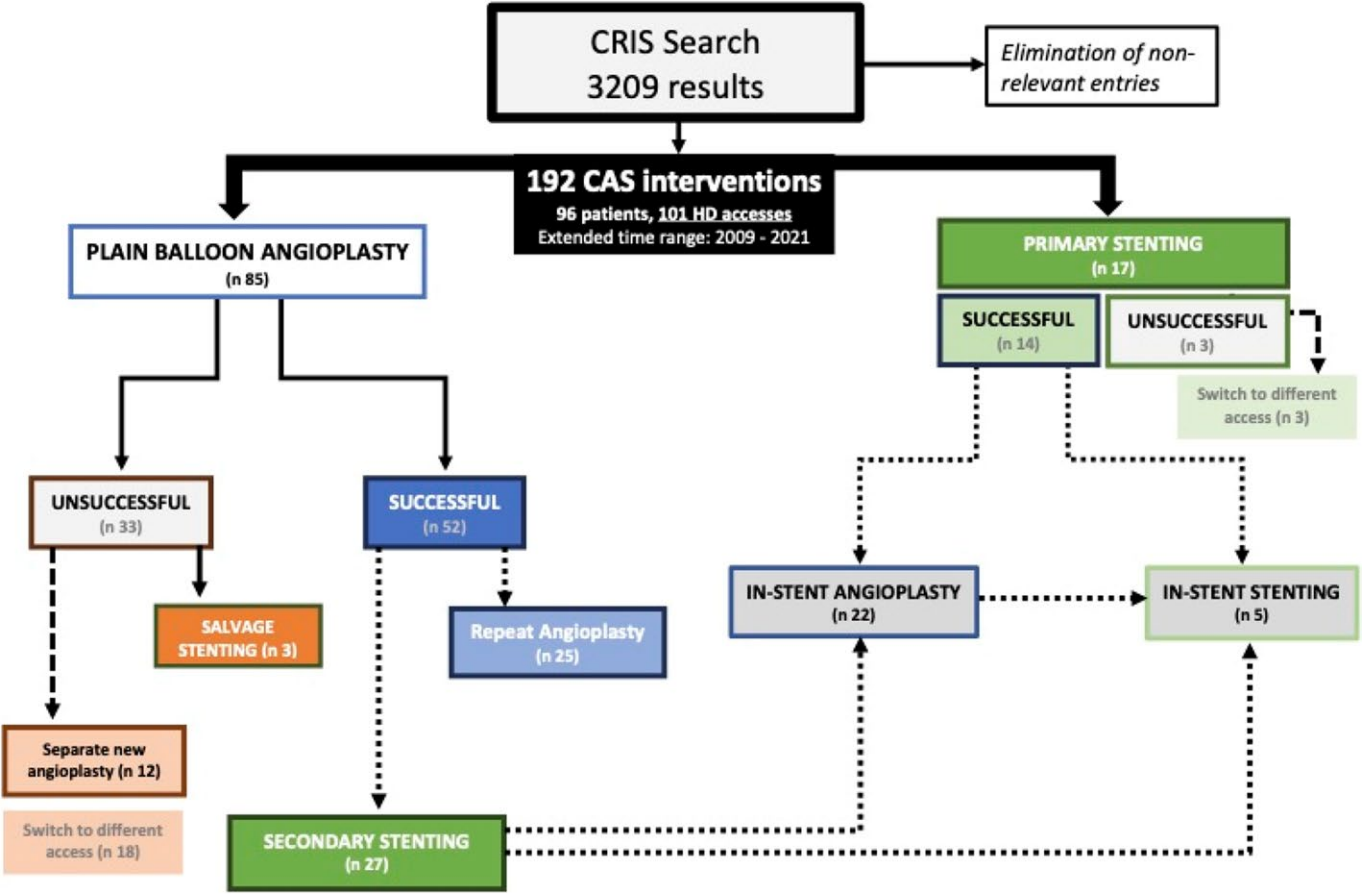
Flowchart of the study

**Fig. 3:**
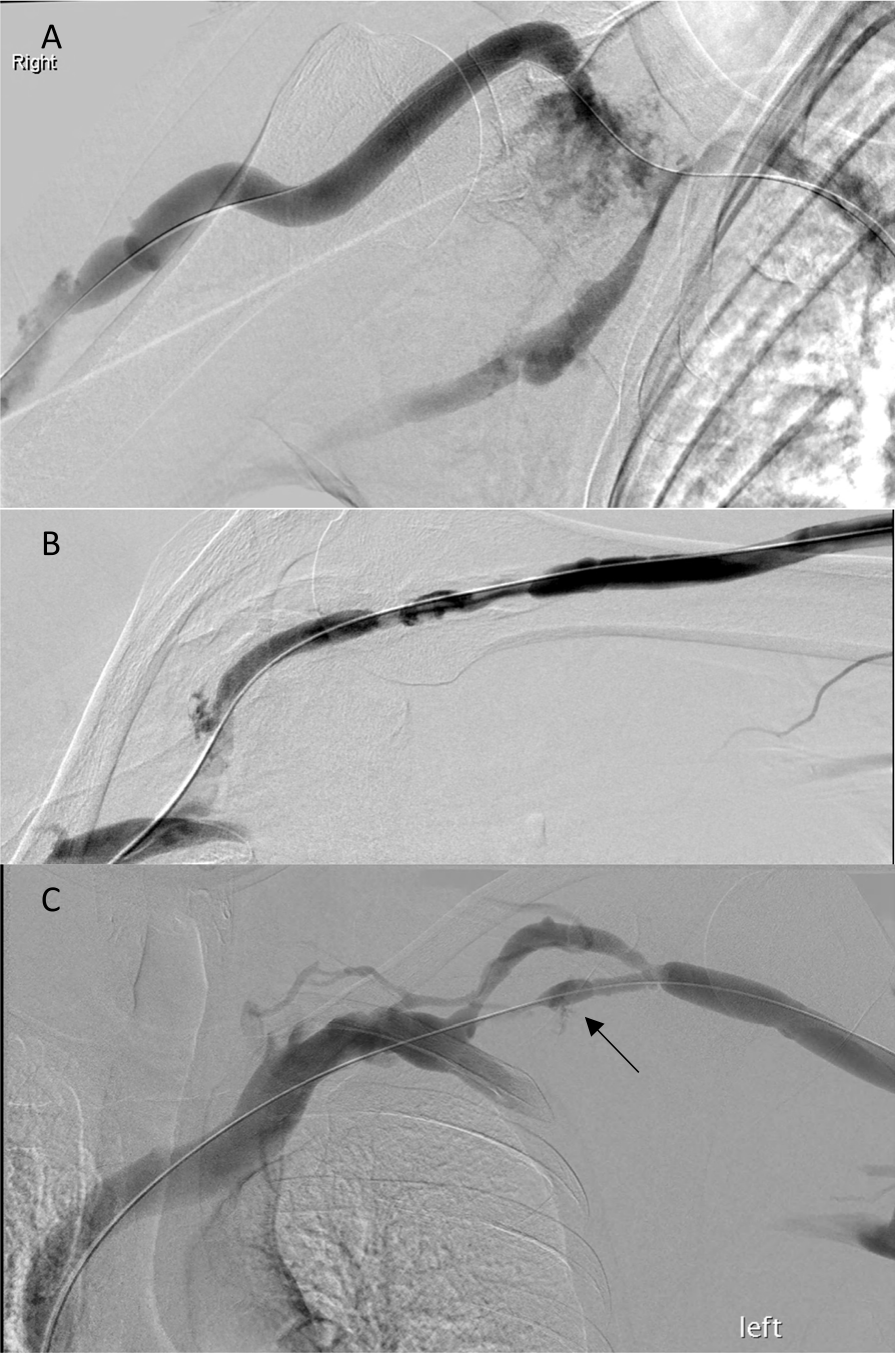
Rupture cases of different radiological severity in study population. **A** Conspicuous haemorrhage post-PTA. **B** Mild to moderate extravasation from upper aspect of CA. **C** Subtle iatrogenic injury after angioplasty of lower moiety of a duplicated CA (arrow)

*Technical success was obtained in 52 / 85 of primary PBA (61.2%).* A persistent stenosis in a failed primary angioplasty in those 33 cases would have prompted the operator to perform a second balloon deployment, deploy a stent, or accept the results and recommend short follow-up imaging, with view to repeat angioplasty or possible stenting; in 18 cases the HD access was abandoned altogether (Fig. 2). Venous rupture (Fig. 3) was noted 9 times (10.6%) of primary PTA and managed with prolonged inflation (6 cases) or salvage SG placement (3).

### Stenting

**Fig. 4:**
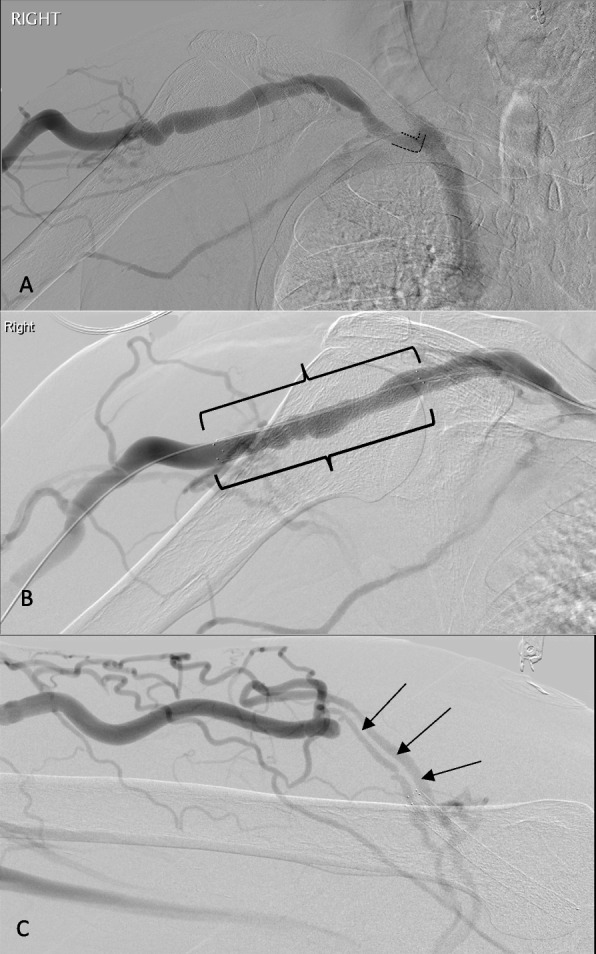
**A** A CAS in the medial segment *(domain C)* [i.e., confluence with the subclavian vein] was described on a diagnostic fistulogram, despite the suboptimal view (*dotted lines*). **B** The patient underwent expedited SG insertion, but the operator erroneously stented the tortuous lateral aspect of the CA (*braces*). **C** The cephalic conduit was found to be occluded from the level of the shoulder after 4 weeks from SG insertion (*black arrows*)

In 45 SG insertions and 4 in-stent stenting procedures a self-expanding PTFE-covered SG with nitinol frame devices was used (*Viabahn*, W.L. Gore & Associates Inc., Flagstaff, AZ, USA); a *Covera* vascular covered stent (Bard, New Jersey, USA) was used in 2 stenting and 1 in-stent stenting cases. 19 SG (40.5% of stenting procedures) were deployed from the CA to lie in the central vein (i.e., ≥ 1 cm within subclavian vein); the remainder were deployed at the ostium of the deep draining vein. No ruptures occurred, with technical success in first intervention (17/20, 85%) and overall majority (*n* = 44, 93.6%). In two cases, > 30% residual stenosis persisted even after balloon moulding, and in one case, the ascending segment was erroneously stented instead of the confluence, leading to thrombosis of the whole conduit shortly after (Fig. 4). No long-term untoward effects of SG deployment (i.e., flow reduction or occlusion of the deep veins, infection, shoulder movement impingement, SG migration) were observed in the study population.

### Univariate analysis

From an individual intervention basis, we differentiate two types of stenting: insertion of a SG in the CA as first intended modality of treatment, defined as *primary elective*. On the contrary, *salvage stenting* refers to SG insertion after poor response to PBA, or emergency stenting after venous rupture, during the same intervention. *Secondary* stenting instead refers to the placement of a SG as later intervention during the history of the access (Fig. 2).

Elective and salvage stenting combined (*n* = 20), compared to first angioplasty, had lower rupture rates (no cases vs. 9/85, 10.6%, Fisher’s *p* = 0.046), and higher technical success (17/20, 85% vs. 52/85, 61.2%; Fisher’s, *p* = 0.003; Table 3). There was no difference in dialysis efficacy (URR) before and after intervention (Wilcoxon, *p* = 0.92); however, 17 accesses undergoing CAS treatment mainly for poor URR (< 65%) (8 angioplasties, 6 primary stentings, 3 salvage stentings) had an average pre-intervention value URR of 55 (SD ± 11.9; median 61, IQR 50–63.5) and a post-intervention of 67 (SD ± 7.3; median 67, IQR 62–71), resulting in a significant improvement (Wilcoxon, *p* = 0.002).

**Table 3:** Crosstabulation of angioplasty and stenting interventions in relation to success and rate of venous rupture. * Fisher’s exact test

**Overall technical success (<30% STENOSIS)**		
PTA	SG	*p*
52 / 85 (61.2%)	17 / 20 (85%)	*0.003*
**Rupture**		
angioplasty	stenting	*p*
9 / 85 (10.6%)	0 / 20 (0%)	*0.046

**Fig. 5:**
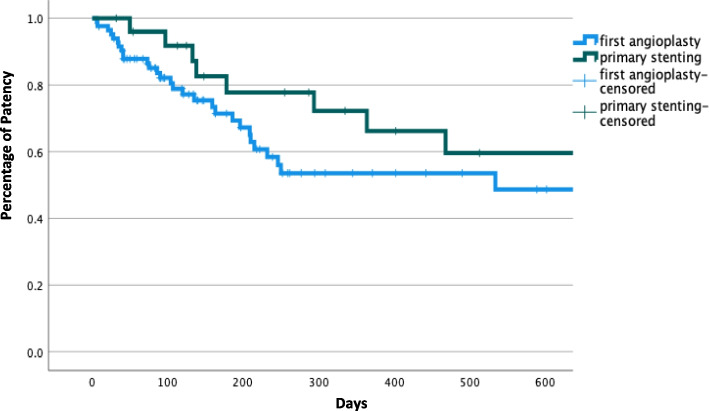
Kaplan-Meier to assess primary patency (PP) between primary elective and salvage stenting (n 20) and first angioplasty (n 85) (*p* = *0.195*)

Primary elective SG (*n* = 17) and salvage SG (*n* = 3) had better PP than primary angioplasty (*n* = 85) at 6 months (79% vs 71%), one year (73% vs 51%) and 18 months (60% vs 47%), Log-rank, *p* = 0.195, Fig. 5.

A second PBA (*n* = 25) for recurrent CAS had poorer rates of primary patency (61% at 6 months, and 33% at one year) when compared to the first angioplasty intervention (*n* = 85) (Log Rank, *p* = 0.1).

**Fig. 6:**
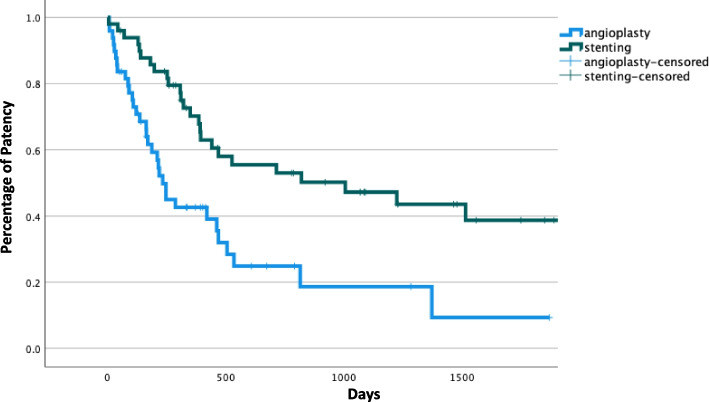
Kaplan–Meier to assess post-intervention assisted primary patency (PAPP) between accesses which received angioplasties (n 54) and accesses which underwent stenting (n 47) at any point during the access history (*p* < *0.001*)

**Fig. 7:**
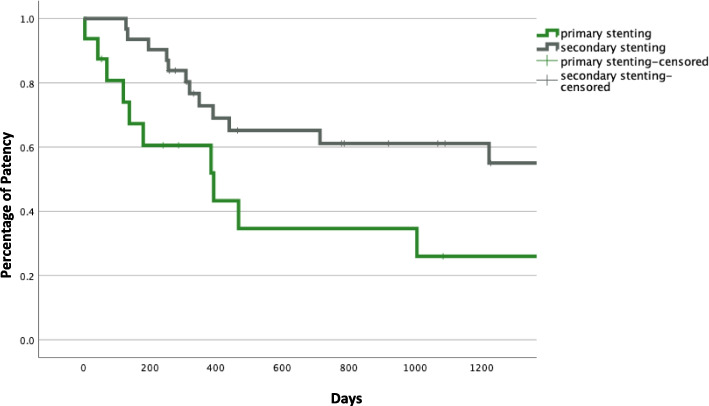
Kaplan–Meier to compare post-intervention assisted primary patency (PAPP) between the two subgroups of primary elective and salvage stenting (n 20) and secondary stenting (n 27) (*p* = *0.010*

HD accesses that underwent SG placement at any point during their history (*n* = 47) had better PAPP or cumulative patency than accesses which underwent repeated PBA only (*n* = 54) (Log Rank, *p* < 0.001), Fig. 6*.* However, the subgroup that underwent primary stenting (elective and salvage, *n* = 20) had significantly inferior cumulative patency than the secondary stenting (*n* = 27) subgroup (60%, 34% and 26% patency at 1, 2 and 3 years; compared to 73%, 61% and 61%) (Log Rank, *p* = 0.010), Fig. 7.

### Multivariate analysis

Technical success was the only association with better outcome on multivariate regression analysis (risk ratio 35%, *p* = *0.035. *Tables 4 and 5) in a model inclusive of the first angioplasty (*n* = 85). For SG, no factors were associated with a better outcome of success (Table 6).

**Table 4:** Proportional hazard Cox regression using intervention-based data on 85 primary angioplasties and 20 primary elective and salvage stenting. Enter model. ^a^For categorical variables, the first category is used as reference

	Regression Coefficient	SE	Hazard Ratio	CI	Significance
Age	- 0.008	0.013	0.981	*0.96 – 1.01*	*0.545*
Sex	0.652	0.360	1.92	*0.947 – 3.8*	*0.07*
Diabetes	- 0.37	0.41	0.69	*0.31 – 1.56*	*0.311*
CA Duplication	0.9	0.49	0.66	*0.25 – 1.73*	*0.84*
25–50% stenosis^a^	- / -	0.49	- / -	- / -	*0.187*
50–75% stenosis	1.65	0.51	1.12	*0.64 – 42.1*	*0.121*
> 75% stenosis	1.85	0.434	6.4	*0.84 – 48.7*	*0.073*
Type A^a^	- / -	0.653	- / -	- / -	*0.237*
Type B	-0.523	0.678	0.6	*0.57 – 2.24*	*0.44*
Type C	0.343	0.648	1.41	*0.396 – 5.1*	*0.064*
Type D	-0.180	0.595	0.835	*0.239 – 2.91*	*0.778*
Angulation between ascending and descending segments *(alpha angle)*	- 0.005	0.009	0.995	*0.98 – 1.02*	*0.607*
Peripheral stenosis	- 0.7	0.382	0.537	*0.254 – 1.3*	*0.104*
central vein stenosis	- 0.002	0.55	0.998	*0.34 – 2.9*	*0.996*

**Table 5:** Proportional hazard Cox regression using only data of 85 primary balloon angioplasties within intervention-based analysis. Enter model. ^a^For categorical variables, the first category is used as reference

	Regression Coefficient	SE	Hazard Ratio	CI	Significance
25–50% stenosis^a^	- / -	1.701	- / -	- / -	*0.168*
50–75% stenosis	1.01	1.116	2.75	*0.327 – 23.1*	*0.35*
> 75% stenosis	1.6	1.051	5.16	*0.364 – 39.9*	*0.17*
Type A^a^	- / -	0.81	- / -	- / -	*0.16*
Type B	-0.051	0.832	0.95	*0.18 – 4.85*	*0.951*
Type C	1.126	0.85	3.1	*0.203—6.9*	*0.18*
Type D	0.196	0.847	1.23	*0.232 – 6.34*	*0.82*
Peripheral stenoses	0.580	0.375	1.78	*0.857 – 3.72*	*0.12*
Central vein stenosis	0.288	0.510	1.33	*0.385 – 2.839*	*0.57*
Balloon Size	-0.005	0.128	0.968	*0.781 – 1.267*	*0.964*
**Technical success**	**-1.056**	**0.45**	**0.35**	*0.147—0.842*	***0.035***

**Table 6:** Proportional hazard Cox regression using data from 20 interventions of primary elective and salvage stenting. Enter model. For categorical variables, the first category is used as reference

	Regression Coefficient	SE	Hazard Ratio	CI	Significance
Stent diameter	-1.12	0.957	0.751	*0.342 – 1.225*	*0.247*
Stent length	3.41	0.008	1.01	*0.993 – 1.025*	*0.27*
Angulation of CA after stent deployment	-0.015	0.01	0.985	*0.966 – 1.024*	*0.71*
Deployment into central veins	-0.058	0.856	0.943	*0.176 – 5.05*	*0.94*
S/V Ratio	0.31	1.227	1.36	*0.045 – 41.35*	*0.86*

## Discussion

Despite the multiple reports of the superiority of stents and SG in treating lesions at the CA [28, 29], PTA continues to be commonly used as a first line intervention in many centres due to much lower cost [18, 20, 24, 25]. Bearing in mind that HD accesses are usually subject to regular clinical and radiological surveillance, this balloon-first approach was also non-detrimental in our patient population: the observed reduced PP when PTA is not technically successful has been reported elsewhere [30].

While SG are regarded as effective, safe, and better than PBA in CAS [28, 29], the long-term patency of SG remains limited: the subgroup of patients in whom a SG was inserted primarily displayed significantly worse cumulative patency: this might be attributable to the pre-existing unmeasured characteristics of this subgroup of accesses (e.g., underappreciated critical luminal diameter on conventional angiography; or unfavourable flow rates [6]), or simply the small size of this subgroup, rather than the stenting intervention per se.

In our patients, SG diameters, apposition, S/V ratio, had no impact on outcome, while there have been reports of better patency by deploying undersized stents into the subclavian vein [31] instead of positioning them flush with the subclavian ostium (the conventional way [6]). The latter technique was the preference of this unit (without breaching with a SG into the deep system when possible), thus we were unable to ascertain if this was a factor in determining outcome. Also, the relatively small size of the subgroups limits the capacity to detect significant predicting variables.

Information on access *Qa* and minimal luminal diameter (MLD) of CAS [6] are of great importance when directing individual therapy but clinical practice may relate more to the efficacy of dialysis parameters. While acknowledging the limitations of a standard two-dimensional angiography, production of the optimal views of the CA would require dedicated positioning the C-arm caudocranial and with contralateral anterior-oblique angulation (e.g., 10-20º CC and 5-10º AO). It is also valuable to acquire images of the CA prior to crossing it with a wire, to avoid underestimating a stenosis which may be masked by artificial wire-induced straightening. Equivocal images and complex multifocal lesions that are deemed to benefit from multidisciplinary approach are discussed at our institution on a weekly basis.

Once a CAS is confirmed, stenting might be inevitable during the time of the access. However, we have never encountered interim access thrombosis in situations when the operator deferred stenting to allow a new expedited discussion of the clinical findings and angiographic images at the multidisciplinary meeting. For this reason, while we have a low threshold for SG insertion, we continue to regard PBA as a safe and reliable first approach in many cases of CAS; acknowledging though that reintervention with associated costs and impact on patients, is likely. On the contrary, a second PBA to address recurrent CAS is less likely to be beneficial and we tend to avoid it altogether.

### Limitations

This study suffers from the conventional limitations of a retrospective data collection. In this regard, a few patients after the first unsuccessful angioplasty were not offered a secondary stenting; analysis of this subgroup might have been valuable. To increase data homogeneity, small numbers of interventions with other types of balloons (cutting, DCB, scoring, high-pressure) apart from PBA were excluded. Arteriovenous grafts and BMS interventions were excluded by the same rationale. A comprehensive statistical adjustment for the variables could not be performed due to uneven subgroups sizes. As it is the case of most other studies, data related to Qa and MLD were not collected, and we did not routinely measure flows before and after treatment. PTA and SG were deployed using conventional angiography, not intravascular US nor cone-beam CT. We also acknowledge that the available clinical information is related to technical success: estimation of the clinical success is primarily reflected by data on patency and URR.

## Conclusion

Several therapeutic armaments are available in the context of the failing arteriovenous access, but CAS remains a difficult scenario to address: peripheral or central stenoses do not have a comparably unfavourable prognosis in our experience. Our data confirm that SG are superior in treating lesions at the cephalic arch, in particular when there is no response following balloon. A stented HD access has better longevity thanks to patency-assisting procedures, but when to stent remains a matter of debate: an angioplasty can successfully address a CAS with improvement of dialysis performance. When clinically appropriate, inserting a SG at later stage after PTA rather than primarily did not negatively affect access longevity in our patient data: a ‘balloon-first’ approach remains the preferred strategy in our unit in most cases.

Aside from the dichotomy between angioplasty and stenting, other variables related to inflow, ouflow, lesion and conduit characteristic play a role at determining the arteriovenous access patency. And while some factors and predictors may be hard to determine, we find that complex CA appearances always benefit from a comprehensive and constructive multidisciplinary discussion, at no detriment to patients’ wellbeing and haemodialysis access.

## Data Availability

Data can be made available from the corresponding author on reasonable request.
